# Antimicrobial resistance surveillance of *Escherichia coli* from chickens in the Qinghai Plateau of China

**DOI:** 10.3389/fmicb.2022.885132

**Published:** 2022-07-22

**Authors:** Biao Tang, Jingge Wang, Xue Zheng, Jiang Chang, Jiangang Ma, Juan Wang, Xiaofeng Ji, Hua Yang, Baoan Ding

**Affiliations:** ^1^State Key Laboratory for Managing Biotic and Chemical Threats to the Quality and Safety of Agro-products, Institute of Agro-product Safety and Nutrition, Zhejiang Academy of Agricultural Sciences, Hangzhou, China; ^2^College of Agriculture and Animal Husbandry, Qinghai University, Xining, China; ^3^State Key Laboratory of Microbial Metabolism, School of Agriculture and Biology, MOST-USDA Joint Research Center for Food Safety, Shanghai Jiao Tong University, Shanghai, China; ^4^College of Veterinary Medicine, Northwest A&F University, Yangling, China

**Keywords:** *Escherichia coli*, antimicrobial resistance, Qinghai Plateau, genome sequences, *mcr-*1

## Abstract

Antimicrobial resistance (AMR) may lead to worldwide epidemics through human activities and natural transmission, posing a global public safety threat. Colistin resistance mediated by the *mcr-*1 gene is the most prevalent among animal-derived *Escherichia coli*, and *mcr-*1-carrying *E. coli* have been frequently detected in central-eastern China. However, animal-derived *E. coli* with AMR and the prevalence of *mcr*-1 in the Qinghai Plateau have been rarely investigated. Herein, 375 stool samples were collected from 13 poultry farms in Qinghai Province and 346 *E. coli* strains were isolated, of which eight carried *mcr-*1. The AMR rates of the *E. coli* strains to ampicillin, amoxicillin/clavulanic acid, and tetracycline were all above 90%, and the resistance rates to ciprofloxacin, cefotaxime, ceftiofur, and florfenicol were above 70%. Multidrug-resistant strains accounted for 95.66% of the total isolates. Twelve *E. coli* strains showed colistin resistance, from which a total of 46 AMR genes and 36 virulence factors were identified through whole-genome sequencing. The *mcr*-1 gene resided on the IncHI2, IncI2-type and IncY-type plasmids, and *mcr*-1 was located in the *nikA-nikB-mcr-*1-*pap2* gene cassette (three strains) or the *pap2-mcr-*1-IS*Apl1* structure (one strain). Completed IncI2-type plasmid pMCR4D31–3 sequence (62,259 bp) revealed that it may cause the horizontal transmission of *mcr-*1 and may increase the risk of its spread through the food chain. Taken together, the AMR of chicken-derived *E. coli* in the plateau is of concern, suggesting that it is very necessary for us to strengthen the surveillance in various regions under the background of one health.

## Introduction

With rising antimicrobial resistance (AMR) to less toxic antibiotics, colistin is used as a backup drug to treat infections caused by carbapenem-resistant Enterobacteriaceae pathogens (Tonny and Anti, 2015; Sarica and Yurttutan, 2018). Due to the unregulated use of colistin in livestock before the antibiotics ban, colistin-resistant Enterobacteriaceae have already become prevalent. The gene *mcr-*1(1,626 bp), which mediates colistin resistance, was first reported on the IncI2-type plasmid of *Escherichia coli* (SHP45; Liu et al., 2016). Subsequently, the *mcr* gene series (*mcr-*1 to *mcr-*10) that mediates colistin resistance in plasmids of enterobacteria has been successively reported in more than 50 countries (Ling et al., 2020; Yang et al., 2020; Pan et al., 2021; Xu et al., 2021). The *mcr-*1 gene is present in various types of *E. coli* plasmids, including IncI, IncX, IncHI, IncP, and IncF (Wang et al., 2017). Of these, IncI2, IncX4, and IncHI2 are common and were the earliest reported plasmid types carrying the *mcr-*1 gene. The IncHI2-type plasmids from different origins are similar to each other and have become the most common effective carrier for the spread of *mcr-*1 in humans, animals, and food (Chang et al., 2020).

*Escherichia coli* is a common pathogenic bacteria in human infections and has caused various medical and community public safety issues. Manges et al. retrospectively analyzed relevant studies from 1995 to 2018 and found that the epidemiology and population dynamics of *E. coli* are complex and dynamic (Manges et al., 2019). It is worth noting that *E. coli*, as an indicator of the prevalence of Gram-negative bacteria, acts as a rich antibiotic resistance gene pool and a mobile center of resistance gene exchange (Poirel et al., 2018). The high prevalence of *mcr-*1, a colistin resistance gene in *E. coli*, poses a clinical threat, and its diverse genetic mechanisms have jointly promoted its spread and long-term presence (Wang et al., 2018; Zhong et al., 2018). China and Vietnam are the two countries with the most *mcr-*1-positive isolates verified through genome sequencing (Wang et al., 2018). However, studies on *mcr-*1 in China have been mostly concentrated in the central, eastern, and southern regions, while data in the northwestern region, especially in Qinghai Province, are still limited. This disparity may be related to the high altitude, low population density, and low avicultural intensity of Qinghai Province, allowing for poultry farming to be kept away from other human activities in most areas in this region. In the past, studies related to animal-derived *mcr-*1-carrying *E. coli* in Qinghai included four *E. coli* strains isolated from sheep diarrhea samples (Tang et al., 2019b), 14 *E. coli* strains isolated from pig feces specimens, and six extended-spectrum beta-lactamases (ESBLs)-carrying *E. coli* strains found in pigs and yaks in Xining and Haidong, respectively (Tong et al., 2018; Wang et al., 2020). Of the above-described resistance gene-carrying *E. coli* strains, only the sheep-derived *E. coli* strain has been fully genome sequenced. Moreover, animal-derived drug-resistant *E. coli* strains in this region have rarely been reported, and the scattered published results are insufficient for comprehensively understanding animal-derived AMR in Qinghai Province and its risks.

Surveillance of AMR in China is mainly in developed provinces and rarely involved in remote areas. Qinghai Province represents a unique geographical unit and is located in the hinterland of the Qinghai-Tibet Plateau, which is known as the third pole of the earth and the roof of the world. As one of the areas in China least disturbed by human activities, Qinghai is the origin of three rivers, i.e., the Yangtze River, the Yellow River, and the Lancang River, and has rich biodiversity (Wu et al., 2016). From 2012 to 2013, an *E. coli* strain obtained from wild *Marmota himalayana* in Yushu, Qinghai, was found to be sensitive to 23 antibiotics including penicillins, cephalosporins, and carbapenems (Bai et al., 2016; Lu et al., 2016). However, all *E. coli* strains obtained from wild plateau pika (*Ochotona curzoniae*) in Qinghai Province in 2016 were penicillin-resistant and some strains were cephalosporin- and streptomycin-resistant (Bai et al., 2016). Therefore, the evaluation of the AMR of zoonotic *E. coli* in Qinghai Province can help us understand the status of antibiotic pollution in animal husbandry and apicultural environments and provide references for formulating animal husbandry and natural environmental protection policies in this unique area.

In this study, we collected fresh stool samples from chickens of different varieties and different ages under different farming environments in Qinghai Province (at altitudes of ~2,500–4,000 m) from June 2020 to July 2021 and analyzed drug-resistant *E. coli* strains in the samples to address the inadequacy of previous studies on drug-resistant bacteria of animal origin in Qinghai Province. We performed whole-genome sequencing on the colistin-resistant *E. coli* isolates to reveal the prevalence of the animal-derived AMR gene *mcr-*1 and its transmission patterns in the environment.

## Materials and methods

### Isolate information

**Figure 1:**
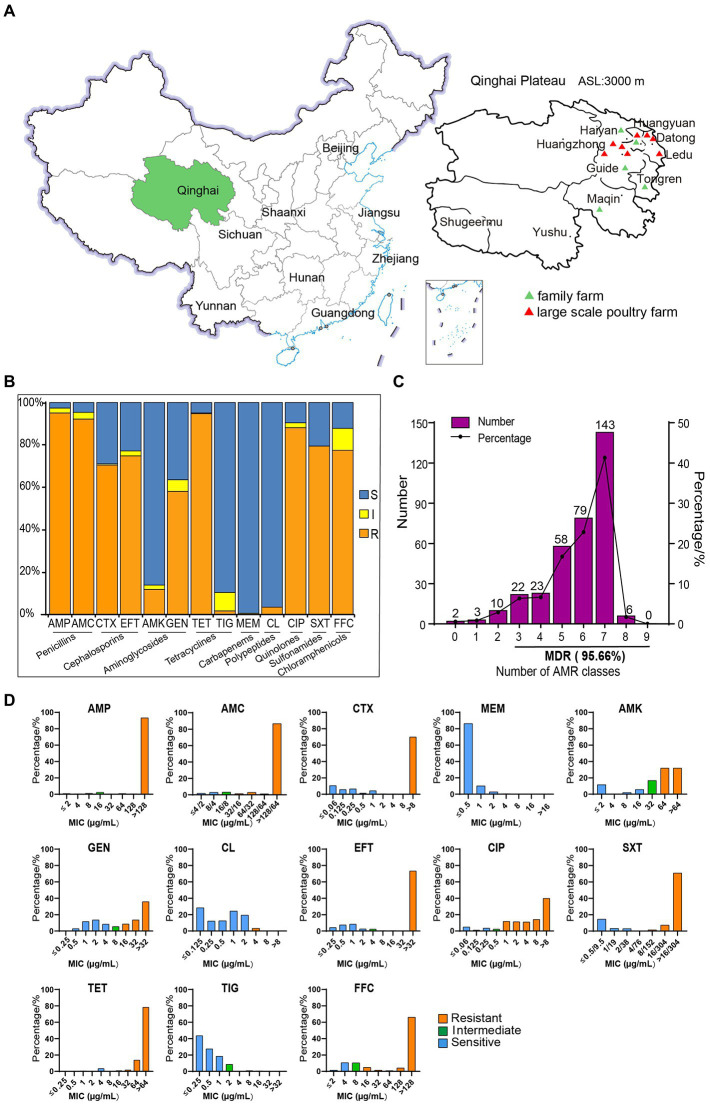
The sampling locations and susceptibility test results of 346 strains of *Escherichia coli* to 13 antibiotics. **(A)** Sampling areas in Qinghai Province, China. **(B)** AMR rates of *E. coli*. **(C)** The distribution of multidrug-resistant strains. **(D)** MIC distributions of 13 antibiotics in isolated *E. coli* strains.

From June 2020 to July 2021, 375 cloaca stool samples were collected from 13 chicken farms in Qinghai Province (Figure 1A; Supplementary Table 1). Then, they were cultured in 5 ml of sterile buffered peptone water (BPW) at 37°C for enrichment, and then sequentially cultured in MacConkey agar (MacConkey, MAC) and eosin methylene blue (EMB) agar medium for identification. Putative *E. coli* colonies were selected and cultured on Luria broth (LB) plates and purified through three rounds of streaking, from which single *E. coli* colonies were determined through Bruker time-of-flight mass spectrometry (MALDI-TOF MS, Germany; Kostrzewa, 2018; Tang et al., 2021). The identified *E*. *coli* strains were stored at −80°C in a freezer in a 25% glycerol solution.

### Antimicrobial susceptibility testing

All isolated strains of *E. coli* were tested for antibiotic resistance using the standard broth dilution method according to the guidelines of the Clinical and Laboratory Standards Institute (CLSI). The minimal inhibitory concentration (MIC) of 13 antibiotics was detected using a drug susceptibility panel for Gram-negative aerobic bacteria. The panel of antimicrobial compounds tested included ampicillin (AMP; 2–128 μg/ml), amoxicillin/clavulanate potassium (AMC; 4/2–128/64 μg/ml), cefotaxime (CTX; 0.06–8 μg/ml), meropenem (MEM; 0.5–16 μg/ml), amikacin (AMK; 2–64 μg/ml), gentamicin (GEN; 0.25–32 μg/ml), colistin (CL; 0.125–8 μg/ml), ceftiofur (EFT; 0.25–32 μg/ml), ciprofloxacin (CIP; 0.06–8 μg/ml), sulfamethoxazole (SXT; 9.5–304 μg/ml), tetracycline (TET; 0.25–64 μg/ml), tigecycline (TIG; 0.25–32 μg/ml), and florfenicol (FFC; 2–128 μg/ml; Tang et al., 2020).

To determine the bacterial viability of *E. coli*, serially diluted mid-log cultures were spotted on LB plates supplemented with different concentrations of colistin (0, 0.25, 0.5, 1, 2, 4, 8, and 16 μg/ml) and incubated at 37°C for 12 h. The *E. coli* strain ATCC 25922 was used as a negative control (Tang et al., 2019a).

A MIC test strip (Liofilchem^®^, Italy) was used to detect the MIC value of colistin-resistant *E. coli* and strain ATCC 25922 in this experiment. A sterile cotton swab was immersed in 0.5 McFarland standard suspension, which was then spread evenly on the MH (OXOID, England) agar medium (Tang et al., 2019a; Chang et al., 2020).

### Whole-genome sequencing, identification of antimicrobial resistance genes, multilocus sequence typing analysis, virulence factors, and plasmid replicon types

DNA was extracted from strains of *E. coli* isolates showing colistin resistance using a commercially available bacterial genomic DNA isolation kit (Generay, China), according to the manufacturer’s instructions. The concentration of DNA in the extracted samples was detected with a NanoDrop 2000 spectrophotometer (Thermo Fisher, United States). The presence of the *mcr*-1 gene in *E. coli* isolates was detected by amplification using *mcr*-1-specific primers (Chang et al., 2020).

Indexed Illumina sequencing libraries were prepared using a TruSeq DNA PCR-free sample preparation kit (Illumina Inc., San Diego, CA) following the standard protocol, and samples were sequenced on the Illumina NovaSeq 6,000 platform according to the manufacturer’s protocols, thus producing 150-bp paired-end reads. The sequence was assembled with a CLC Genomics Workbench 12. Libraries of genomic DNA of representative strain were prepared by SQK-LSK109 kit (Oxford Nanopore Technologies, United Kingdom) and sequenced using R9.4 flow cell technology on a GridION sequencer. The reads were assembled using Unicycler 0.4.8. The NCBI Prokaryotic Genome Annotation Pipeline was used for gene prediction and annotation of the genome.

Antimicrobial resistance genes were predicted using ResFinder 4.1 software (Zankari et al., 2012).^1^ Virulence factors were predicted using Virulence Finder 2.0 software^2^ and the virulence factor database.^3^ Plasmid replicon types were predicted using PlasmidFinder 2.1 software.^4^ Multilocus sequence typing (MLST) was carried out as previously described using MLST 2.0 software.^5^ The BLAST Ring Image Generator (BRIG) and Easyfig software were used in the comparative analysis of plasmids (Alikhan et al., 2011; Sullivan et al., 2011).

### S1-pulsed-field gel electrophoresis, Southern blotting, and conjugation transfer

The *Salmonella enterica* serovar Braenderup H9812 (as size marker) and *E. coli* cells harboring the *mcr-1* gene were fixed by SeaKem Gold Agarose (Lonza Group AG, United States) and subsequently lysed. The embedded DNAs were digested using *Xba* I or S1 nuclease (Takara Bio, China) in a 37°C water bath for 3 h. The restricted DNA fragments were separated in 0.5 × Tris-Borate-EDTA buffer (Sangon Biotech, Shanghai, China) at 14°C for 18 h using a pulsed-field electrophoresis system (CHEF Mapper, Bio-Rad, United States) with pulse times of 2.2 to 63.8 s. The gel block was stained and observed with a gel imager (Tang et al., 2019a; Liang et al., 2021b). The *mcr-1*-specific probe was labeled using a DIG High Prime DNA Labeling and Detection Starter Kit (Roche, Sant Cugat del Vallés, Spain) following the manufacturer’s instructions. *E. coli* J53 was used as the recipient strain, and *E. coli* strains harboring *mcr-1* were used as the donor strain in the conjugation transfer assay. Donor and recipients bacteria were grown together overnight and then inoculated onto LB plates with colistin and sodium azide.

### Statistical analysis

The error values of conjugation transfer efficiency were calculated by GraphPad Prism software. The heatmap of clustering of strains and AMR phenotypes were performed by TBtools. In this analysis, the presence of the above resistance phenotype received a score of 2, intermediate received a score of 1, and the susceptibility received a score of 0.

## Results

### Isolation of *Escherichia coli* and antimicrobial susceptibility

A total of 346 strains of *E. coli* were isolated and identified from 375 stool samples (Figure 1A) collected in Qinghai Province, with an isolation rate of 92.27%. The sensitivity test results of 346 of the *E. coli* strains to 13 antibiotics are shown in Figures 1B,C. In terms of the MIC distribution (Figure 1D), the MIC values of the antibiotics AMP, AMC, CIP, CTX, EFT, TET, and FFC were significantly higher, indicating that the isolated *E. coli* strains are highly resistant to these antibiotics. Among these, AMP, AMC, and TET showed the highest resistance rates of over 90% and were followed by that of CIP, at 88.15%, while CTX, EFT, and FFC exhibited resistance rates greater than 70%. Among the isolates, strains with multidrug resistance (MDR) (resistance to more than three kinds of antibiotics) accounted for 95.66% of the total isolates. The tested strains showed a high resistance rate to GEN (58.09%) and a low resistance rate to AMK (11.85%). In addition, their resistance rates to colistin and TIG were 3.47% and 1.73%, respectively. All of the tested *E. coli* strains were sensitive to MEM. These results indicate that the AMR of chicken-derived *E. coli* in Qinghai Province is serious.

**Figure 2:**
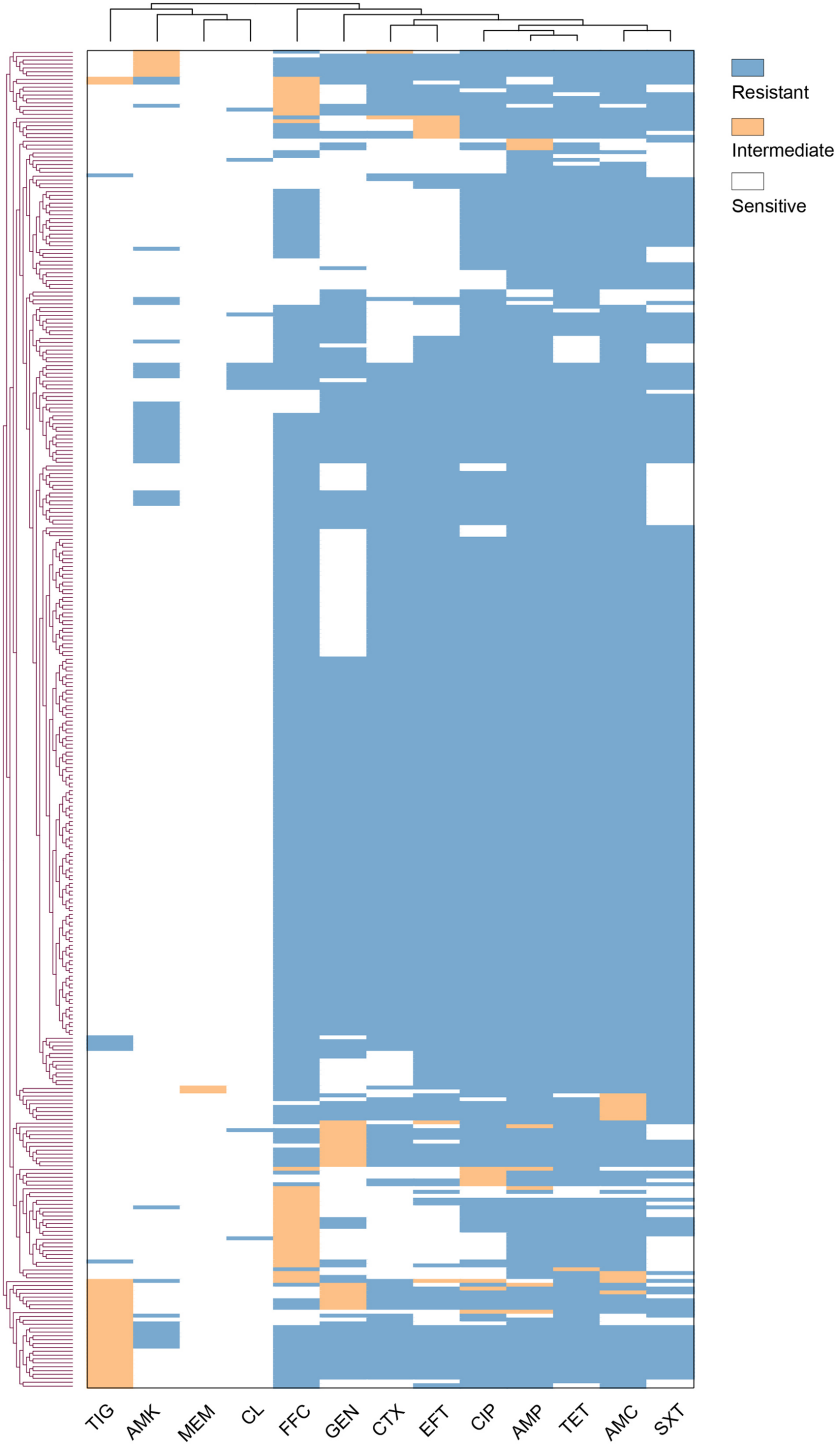
Antimicrobial resistance profiles of 346 *Escherichia coli* strains. Vertical and horizontal trees represent clustering relationships. The abscissa is the antibiotic, and the ordinate is the *E. coli* isolate.

In this study, *E. coli* isolates showed a broad spectrum of resistance to most antibiotics, as shown in Table 1; Figure 2, among which the AMP-AMC-CTX-GEN-EFT-CIP-SXT-TET-FFC-resistant phenotype was the highest proportion (32.08%) out of a total of 111 strains. Among the 346 *E. coli* isolates, only two (0.58%) were sensitive to all of the antibiotics assayed, and the rest exhibited 81 types of resistance across a spectrum. Six of these strains (1.73%) showed resistance to eight of the tested antibiotic categories, with two types of resistance, AMP-AMC-CTX-AMK-GEN-CL-EFT-CIP-SXT-TET-FFC-resistant (four strains) and AMP-AMC-CTX-GEN-CL-EFT-CIP-SXT-TET-FFC-resistant (two strains). One hundred forty-three strains (41.33%) showed resistance to seven of the tested antibiotic categories, with seven types of resistance. Seventy-nine strains (22.83%) showed resistance to six of the tested antibiotic categories, with 18 types of resistance. In the multidrug resistance assay, 95.66% of the isolates showed resistance to the 13 tested antibiotics, indicating that in Qinghai Province, multidrug-resistant chicken-derived *E. coli* strains are common and have diverse and wide AMR spectra.

**Table 1:** Antimicrobial resistance profiles of 346 *Escherichia coli* strains.

ID	Antibiotic resistance pattern	Number	Percentage (%)
1	AMP-AMC-CTX-GEN--EFT-CIP-SXT-TET-FFC	111	32.08
2	AMP-AMC-CTX-EFT-CIP-SXT-TET-FFC	40	11.56
3	AMP-AMC-CIP-SXT-TET-FFC	21	6.07
4	AMP-AMC-CTX-AMK-GEN-EFT-CIP-SXT-TET-FFC	19	5.49
5	AMP-AMC-CTX-EFT-CIP-TET-FFC	7	2.02
6	AMP-AMC-EFT-CIP-SXT-TET-FFC	7	2.02
7	AMP-AMC-SXT-TET	7	2.02
8	AMP-AMC-TET	7	2.02
9	AMP-AMC-CTX-GEN-EFT-CIP-TET-FFC	5	1.45
10	AMP-AMC-GEN-CIP-SXT-TET-FFC	5	1.45
11	AMP-AMC-CTX-AMK-GEN-CL-EFT-CIP-SXT-TET-FFC	4	1.16
12	AMP-AMC-CTX-AMK-GEN-EFT-CIP-TET-FFC	4	1.16
13	AMP-AMC-CTX-GEN-EFT-CIP-SXT-TET	4	1.16
14	AMP-AMC-CTX-EFT-CIP-SXT-TET	4	1.16
15	AMP-AMC-GEN-EFT-CIP-FFC	4	1.16
16	AMP-AMC-GEN-CIP-SXT-TET	4	1.16
17	AMP-CTX-GEN-EFT-CIP-SXT-TET-FFC	4	1.16
18	GEN-CIP-TET	4	1.16
19	AMP-AMC-CTX-GEN-EFT-CIP-SXT-TET-TIG-FFC	3	0.87
20	AMP-AMC-CTX-AMK-GEN-EFT-CIP-SXT-TET	3	0.87
21	AMP-AMC-CTX-EFT-SXT-TET-FFC	3	0.87
22	AMP-AMC-EFT-CIP-SXT-TET	3	0.87
23	AMP-AMC-CIP-SXT-TET	3	0.87
24	AMP-AMC-CIP-TET-FFC	3	0.87
25	AMP-AMC-CIP-TET	3	0.87
26	AMP-AMC-CTX-GEN-CL-EFT-CIP-SXT-TET-FFC	2	0.58
27	AMP-AMC-GEN-EFT-CIP-SXT-TET-FFC	2	0.58
28	AMP-AMC-CTX-EFT-TET-FFC	2	0.58
29	AMP-AMC-CTX-EFT-CIP-TET	2	0.58
30	AMP-AMC-CTX-EFT-TET	2	0.58
31	AMP-AMC-EFT-CIP-FFC	2	0.58
32	--	2	0.58
33	AMP-AMC-CTX-GEN-CL-EFT-CIP-SXT-TET	1	0.29
34	AMP-AMC-CTX-CL-EFT-CIP-SXT-TET-FFC	1	0.29
35	AMP-AMC-CTX-EFT-CIP-SXT-TET-TIG-FFC	1	0.29
36	AMP-AMC-CTX-GEN-CIP-SXT-TET-FFC	1	0.29
37	AMP-AMC-CTX-GEN-EFT-SXT-TET-FFC	1	0.29
38	AMP-AMC-AMK-GEN-EFT-CIP-SXT-FFC	1	0.29
39	AMP-AMC-GEN-CL-CIP-SXT-TET-FFC	1	0.29
40	AMC-CTX-AMK-GEN-EFT-CIP-SXT-TET	1	0.29
41	AMP-AMC-CTX-CL-EFT-CIP-TET-FFC	1	0.29
42	AMP-AMC-CTX-EFT-CIP-TET-TIG	1	0.29
43	AMP-AMC-CTX-CIP-SXT-TET-FFC	1	0.29
44	AMP-AMC-GEN-EFT-CIP-SXT-FFC	1	0.29
45	AMC-CTX-AMK-GEN-CIP-SXT-TET	1	0.29
46	CTX-AMK-GEN-EFT-CIP-SXT-TET	1	0.29
47	AMP-AMC-CTX-GEN-CIP-TET-FFC	1	0.29
48	AMP-AMC-CTX-GEN-EFT-CIP-TET	1	0.29
49	AMP-CTX-AMK-GEN-EFT-CIP-TET	1	0.29
50	AMP-CTX-GEN-EFT-CIP-SXT-TET	1	0.29
51	AMK-GEN-EFT-CIP-SXT-TET	1	0.29
52	CTX-AMK-GEN-CIP-SXT-TET	1	0.29
53	AMP-GEN-EFT-CIP-SXT-FFC	1	0.29
54	AMP-CTX-EFT-SXT-TET-FFC	1	0.29
55	AMP-CTX-EFT-CIP-SXT-TET	1	0.29
56	AMP-AMC-GEN-CIP-TET-FFC	1	0.29
57	AMP-AMC-GEN-CIP-TET-TIG	1	0.29
58	AMP-AMC-GEN-EFT-CIP-TET	1	0.29
59	AMP-AMC-AMK-CIP-TET-FFC	1	0.29
60	AMP-AMC-AMK-CIP-SXT-TET	1	0.29
61	AMP-AMC-CTX-EFT-SXT-TET	1	0.29
62	AMP-AMC-CTX-EFT-CIP-SXT	1	0.29
63	AMP-AMC-GEN-CIP-FFC	1	0.29
64	AMP-AMC-EFT-CIP-TET	1	0.29
65	AMP-AMC-SXT-TET-FFC	1	0.29
66	AMP-CTX-AMK-GEN-TET	1	0.29
67	AMC-CTX-CIP-TET-FFC	1	0.29
68	CTX-AMK-GEN-SXT-TET	1	0.29
69	AMP-CTX-CIP-TET	1	0.29
70	AMP-AMC-TET-FFC	1	0.29
71	AMP-AMC-CL-TET	1	0.29
72	AMC-CTX-CIP-TET	1	0.29
73	AMP-AMC-EFT	1	0.29
74	AMP-SXT-TET	1	0.29
75	AMP-CL-TET	1	0.29
76	AMP-GEN-TET	1	0.29
77	AMP-AMC-FFC	1	0.29
78	AMC-SXT-TET	1	0.29
79	AMP-AMC	1	0.29
80	AMP-FFC	1	0.29
81	TET	1	0.29
82	SXT	1	0.29

**Figure 3:**
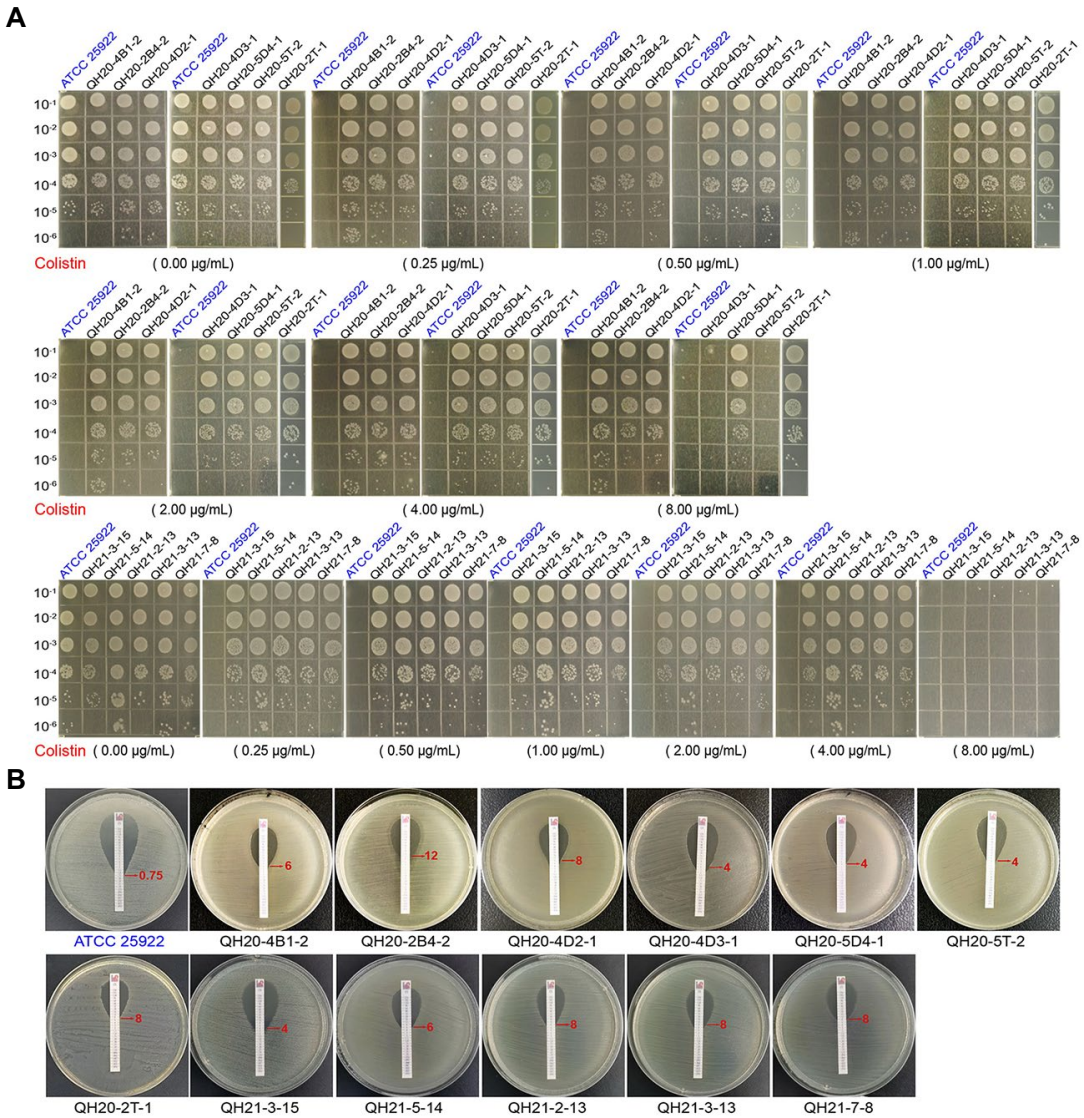
The insusceptibility of *mcr*-1-harboring *Escherichia coli* strains to colistin. **(A)** Bacterial viability of *E. coli* strains on LB plates containing different concentrations of colistin. The diluted suspension (10 μl) was inoculated onto the prepared LB plates. *Escherichia coli* ATCC 25922 was used as a negative control. **(B)** E-test to compare the MIC of colistin in *E. coli* strains. *Escherichia coli* ATCC 25922 was used as a negative control.

Through the agar dilution method (Figure 3A), we found that the control strain ATCC 25922, could grow normally on plates without colistin but could not grow on plates with colistin (≥0.25 μg·ml^−1^). With 8 μg·ml^−1^ of colistin, the growth of QH20-4D3-1, QH20-5 T-2, QH21-3-15, QH21-5-14, QH21-2-13, QH21-3-13, and QH21-7-8 was completely inhibited.

The colistin resistance of eight *mcr-*1-carrying *E. coli* strains was verified with the E-test strip: (0.016–256 μg·mL^−1^ of colistin; Figure 3B). The MIC of the negative control strain ATCC 25922 was 0.75 μg·ml^−1^, while those of the *mcr-*1-carrying *E. coli* strains were all ≥4 μg·ml^−1^, demonstrating colistin resistance.

### Distribution of antimicrobial resistance genes, STs, virulence factors, and plasmid replicon types of *Escherichia coli* isolates

**Figure 4:**
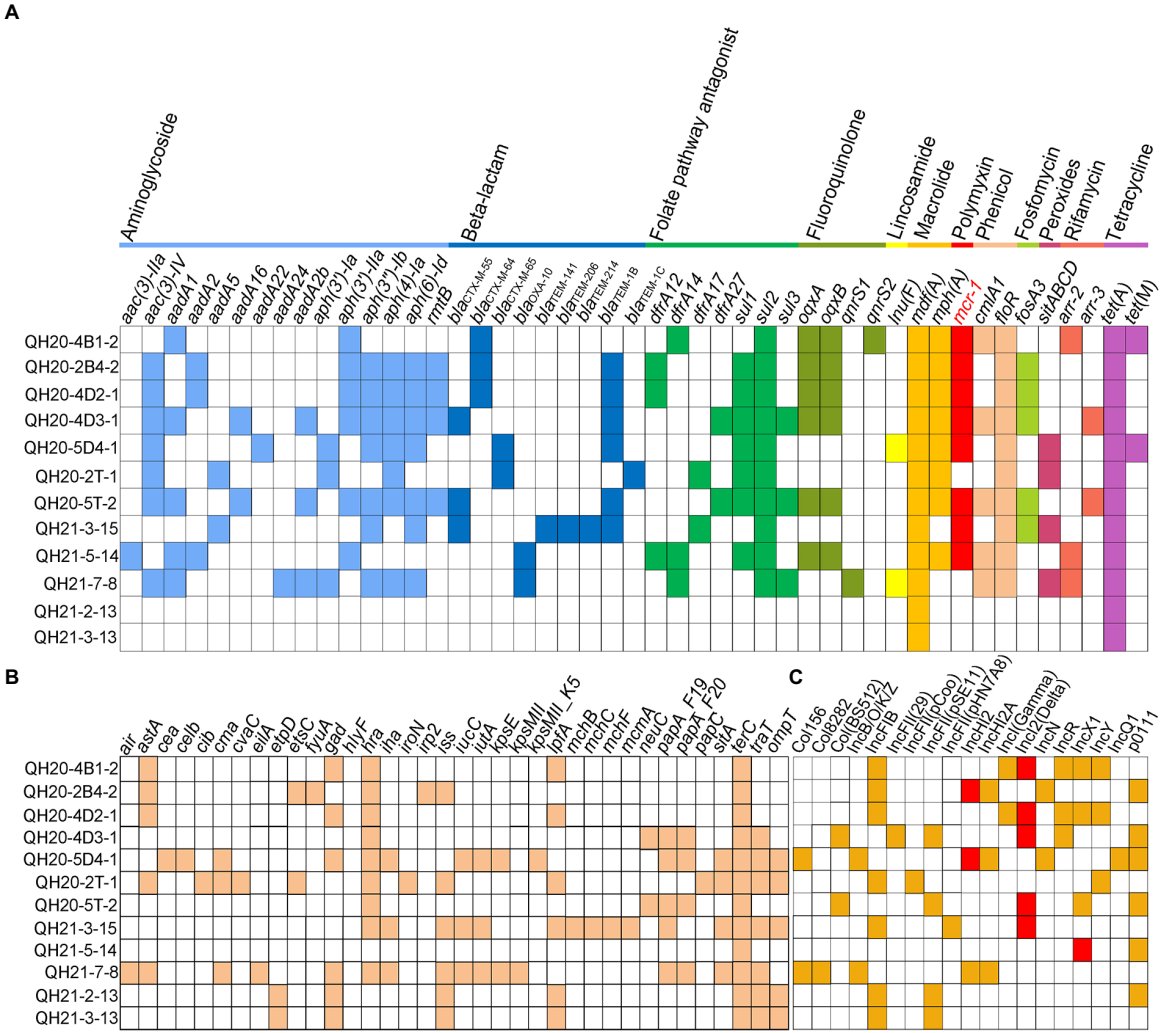
Acquired resistance genes, virulence genes, and plasmid replicon types of *Escherichia coli* strains with whole-genome sequences. **(A)** Antimicrobial resistance (AMR) gene profiles. Various types of AMR genes are labeled with different colors. White squares indicate no AMR genes. **(B)** Virulence gene profiles. Colored squares represent virulence genes, and white squares indicate no virulence genes. **(C)** Plasmid replicon profiles. Colored squares represent the presence of plasmids, and white squares indicate no plasmids.

We sequenced the genomes of all 12 colistin-resistant *E. coli* isolates, from which 46 AMR genes were predicted, as shown in Figure 4A. The complete genome sequence of strain QH20-4D3-1 was performed by third-generation sequencing. Among them, 15 aminoglycoside resistance genes [*aac*(*3*)*-IIa*, *aac*(*3*)*-IV*, *aadA1*, *aadA2*, *aadA5*, *aadA16*, *aadA22*, *aadA24*, *aadA2b*, *aph*(*3′*)*-Ia*, *aph*(*3′*)*-IIa*, *aph*(*3″*)-*Ib*, *aph*(*4*)*-IA*, *aph*(*6*)*-Id*, and *rmtB*], eight beta-lactam resistance genes (*bla*_CTX-M-55_, *bla*_CTX-M-64_, *bla*_CTX-M-65_, *bla*_OXA-10_, *bla*_TEM-141_, *bla*_TEM-206_, *bla*_TEM-214_, *bla*_TEM-1B,_ and *bla*_TEM-1C_), seven folate antagonist pathway resistance genes (*dfrA12*, *dfrA14*, *dfrA17*, *dfrA27*, *sul1*, *sul2*, and *sul3*), four fluoroquinolone resistance genes (*oqxA*, *oqxB*, *qnrS1,* and *qnrS2*), one lincosamide resistance gene [*lnu*(F)], two macrolide resistance genes [*mdf*(A) and *mph*(A)], one peptide resistance gene (*mcr-*1), two phenol resistance genes (*cmlA1* and *floR*), one fosfomycin resistance gene (*fosA3*), two rifampicin resistance genes (*arr-*2 and *arr-*3), and two TET resistance genes [*tet*(A) and *tet*(M)] were detected. These strains were predicted to have nine different ST types (ST29735, ST43, ST10, ST93, ST162, ST2329, ST189, ST4689, and ST259; Supplementary Table 2), indicating that the *E. coli* isolates in this study are highly diverse. 8 of these 12 strains harbored the *mcr-*1 gene.

A total of 36 virulence factors (Figure 4B) and 19 plasmid replicons (Figure 4C) were predicted from the 12 colistin-resistant *E. coli* isolates. Among them, the tellurium ion resistance virulence gene *terC* was found in all 12 strains, and the virulence gene *astA*, which is related to the EAST-1 heat-stable toxin and is a typical marker for entero-aggregating *E. coli* (EAEC), was found in QH20-4B1-2, QH20-2B4-2, QH20-4D2-1, QH20-2 T-1, and QH21-7-8. The plasmid replicators were as follows: Col156, Col8282, Col (BS512), IncB/O/K/Z, IncFIB (AP001918), IncFII (29), IncFII (pCoo), IncFII (pSE11), IncFII (pHN7A8), IncHI2, IncHI2A, INCI (Gamma), IncI2-type (Delta), IncN, INCR, IncX1, IncY, IncQ1, and po111. In the eight *mcr-*1-containing colistin-resistant isolates, the *mcr-*1 gene was located on the IncHI2-, IncI2-, or IncY-type plasmid (Figure 4C). Plasmids carrying the *mcr-1* gene were verified by S1-PFGE and Southern blot (Supplementary Figure 1). The conjugation transfer frequency was ~10^−2^–10^−6^ for four strains QH20-4D2-1, QH21-5-14, QH20-4D3-1, and QH20-5 T-2, with QH20-4D2-1 exhibiting the highest frequency (2.97 ± 0.15) × 10^−2^. Whereas, the *mcr*-1-harboring plasmid in strains QH21-3-15, QH20-4B1-2, QH20-2B4-2, and QH20-5D4-1 were not found to be conjugatively transferred (Supplementary Figure 2).

In QH20-2 T-1, the *etsC, hlyF,* and *ompT* virulence genes all resided on the replicon of the IncFIB-type plasmid. The virulence gene *etpD* was also predicted to be on the IncFIB-type plasmid replicon of QH21-2-13 and QH21-3-13, while the virulence gene *traT* was predicted to be on the IncFII replicon of Plasmid 4 of QH21-3-13 and QH20-4D3-1. The virulence genes *traT* and *ompT* are related to the activity of biofilm outer membrane proteins, *etsC* confers resistance to the serum complement, and the *ets* operon and *ompT* and *hlyF* are related to the conserved virulence region of the FIB replicon (Moran and Hall, 2018). Therefore, different types of plasmid replicons facilitate the horizontal spread of virulence genes.

### Sequence alignment analysis of homologous IncI2-type and IncHI2-type plasmids carrying the *mcr*-1 gene

In different isolates, the contig on which *mcr-*1 resides ranged from 11,011–123,144 bp in size. In QH21-5-14, the contig that contains *mcr-*1 was too short, making it impossible to analyze the sequence. In the other seven *E. coli* isolates, the *mcr-*1 gene was located on the IncI2-type (4 strains) or IncHI2-type (1 strain) plasmid. Here, we selected the sequences of the *mcr-*1-carrying IncI2-type plasmid pHNSHP45 (Acc. No. KP347127) and the IncHI2-type plasmid pHNSHP45-2 (Acc. No. KU341381) that were the earliest to be reported for sequence alignment analysis.

**Figure 5:**
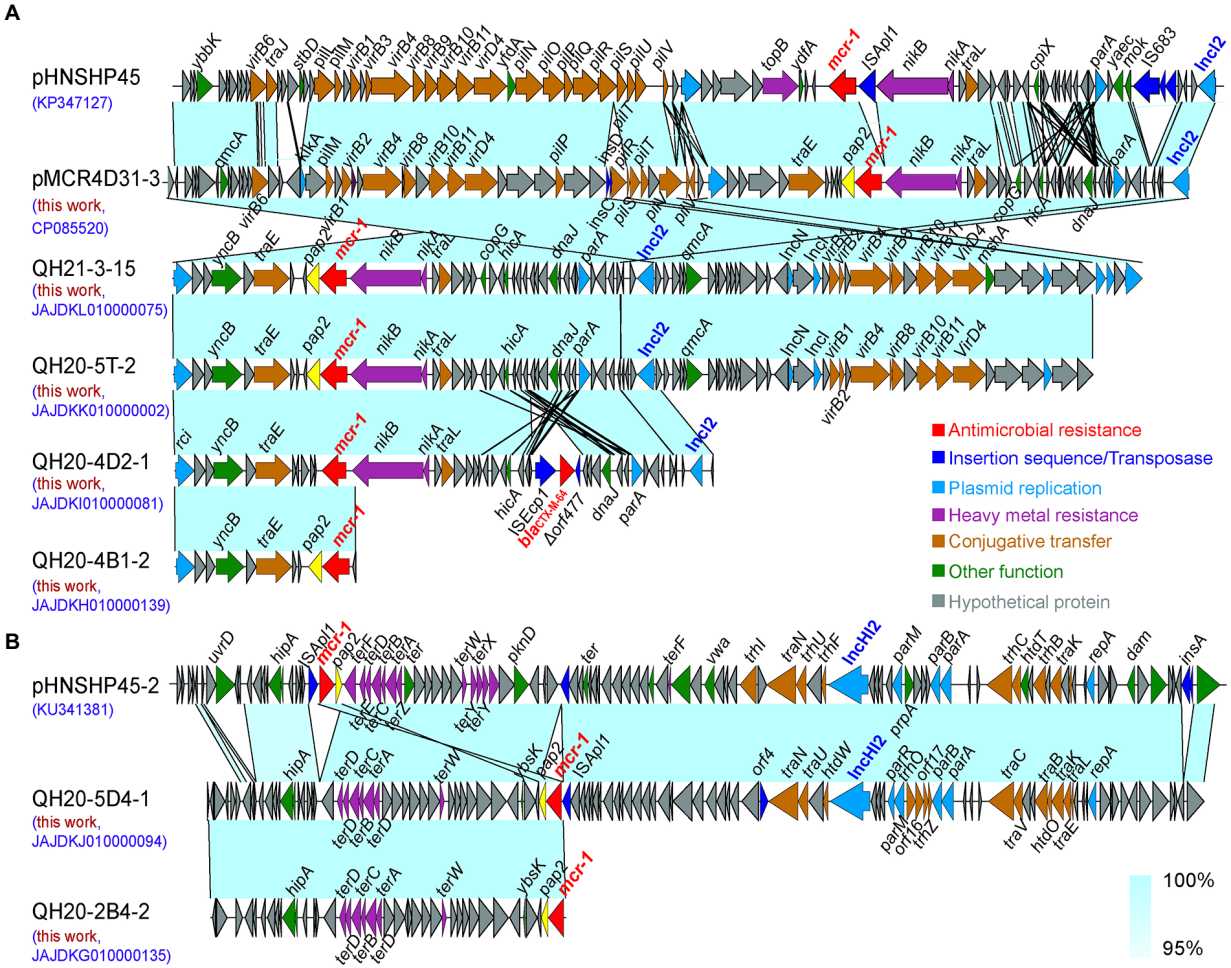
Sequence alignment analysis of IncI2 **(A)** and IncHI2-type **(B)** plasmids carrying the *mcr*-1 gene. The arrows indicate the direction of gene transcription. Functional genes are shown in different colors. Regions of >95% nucleotide homology are indicated by colored shading.

As shown in Figure 5A, the alignment of the IncI2-type plasmid pHNSHP45 sequence with the sequences of plasmids pMCR4D31–3 in QH20-4D3-1, QH21-3-15, QH20-5 T-2, QH20-4D2-1, and QH20-4B1-2 (123.144 kp, GC content: 42.6%, CP085520), QH21-3-15 contig_75 (≈59.073 kp, GC content: 42.2%, JAJDKL010000075), QH20-5 T-2 contig_2 (≈56.045 kp, GC content: 42.2%, JAJDKK010000002), QH20-4D2-1 contig_81 (32.841 kp, GC content: 42.6%, JAJDKI010000081), and QH20-4B1-2 (≈11.011 kp, GC content: 42.3%, JAJDKH010000139) indicates that all sequences were highly homologous, contained the backbone of the typical IncI2-type plasmid, and included the replication-related gene (*repA*), conjugative transfer-related pilus gene cluster genes (*tra*, *pil*), and the plasmid stability-associated gene (*parA*). The backbone region of the IncI2-type plasmid is conserved, containing a shufflon region led by the site-specific recombinase gene (*rci*; Sekizuka et al., 2017). Moreover, upstream of *mcr-*1, all sequences lacked the insertion element IS*Apl1*. The *pap2* gene was always present downstream of *mcr-*1 except for QH20-4D2-1 contig_81 (JAJDKI010000081), which contained *bla*_CTX-M-64_, an independent resistance region of the ESBL resistance gene. This region was located in IS*Ecp1*-*bla*_CTX-M-64_-*Δorf477*, a typical transposon unit, of which the IS*Ecp1* insertion sequence can mediate the transposition of many types of the *bla*_CTX-M_ gene (Zhao et al., 2020). Except in the case of QH20-4B1-2 contig_139 (JAJDKH010000139), the resistance gene *mcr-*1 was integrated at a location downstream to the *nikB* gene, which is identical to that of the majority of *mcr-*1-positive IncI2-type plasmids reported previously. The other five sequences all lacked the IS*683* insertion sequence present in pHNSHP45, while two IS*2* insertion elements were present in the transposase genes *insD* and *insC* downstream of *mcr-*1 in pMCR4D31–3. In pMCR4D31–3, QH21-3-15 contig_75 (JAJDKL010000075) and QH20-5 T-2 contig_2 (JAJDKK010000002), a *virB*/*virD4* secretion system similar to that of pHNSHP45 was present.

As shown in Figure 5B, the sequence alignment of the IncHI2-type plasmid pHNSHP45-2 and QH20-5D4-1 contig_94 (≈107.905 kp, GC content: 44.0%, JAJDKJ010000094) and QH20-2B4-2 contig_135 (≈38.480 kp, GC content: 49.1%, JAJDKG010000135) indicates that the three sequences were highly homologous. In the two IncHI2-type *mcr-*1-containing plasmids obtained in this study, the pHNSHP45-2 toxin-antitoxin system (*higB* and *hipB-A*) and the region mediating resistance to heavy metal tellurium (*terDDCBAD*) were present. QH20-5D4-1 contig_94 (JAJDKJ010000094) carried four partition-associated genes (*parR*, *parM*, *parB,* and *parA*) and 16 plasmid-independent conjugation genes (*traN*, *traU*, *traC*, *traV*, *traB*, *traK*, *traE*, *traL*, *htdO*, *htdW*, *orf4*, *orf17*, *orf16*, *trhP*, *trhO,* and *trhZ*). Moreover, in all cases, the *pap2* gene was present downstream; however, in QH20-2B4-2 contig_135 (JAJDKG010000135), the insertion element IS*Apl1* present in QH20-5D4-1 contig_94 was absent. In the QH20-2B4-2 contig_135 sequence, the 3-terminus of *mcr-*1 was incomplete, and we were therefore unable to obtain information about the genetic elements of plasmid replicon. However, in the QH20-5D4-1 contig_94 sequence, the *pap2-mcr-*1-IS*Apl1* structure that mediates the horizontal transfer of *mcr-*1 and is present in pHNSHP45-2 was observed.

### Analysis of IncI2-type *mcr*-1-carrying plasmids from different sources

**Figure 6:**
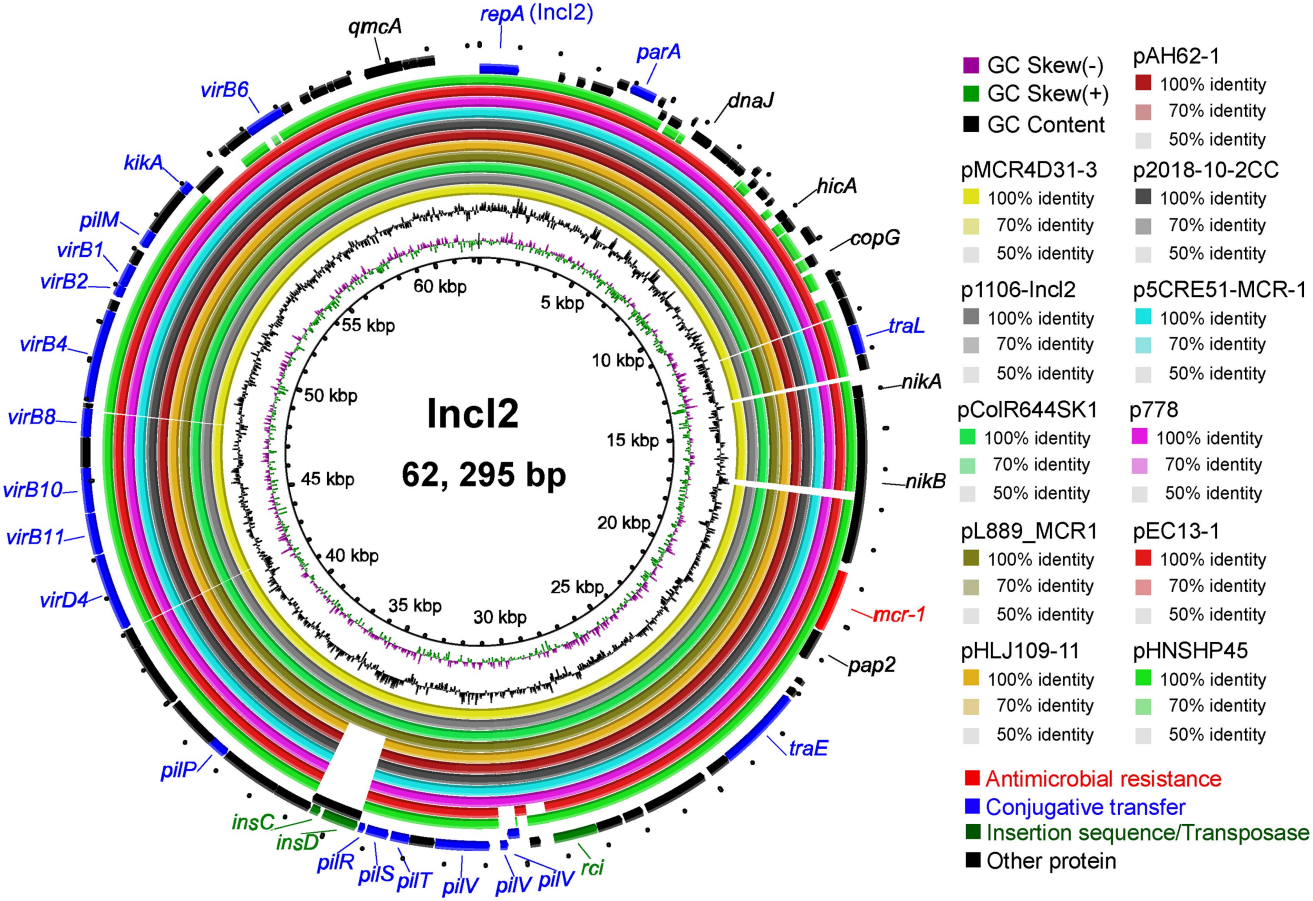
Circular diagram of the IncI2 plasmids. Circles, from inside to outside, indicate the GC skew, GC content, and ORFs in the positive and negative DNA strands. Genes are shown in different colors.

For sequence alignment analysis, we selected nine plasmids from the NCBI database with the highest homology to the sequence of pMCR4D31–3 carried in strain QH20-4D3-1 (Figure 6) and found that the homology between pMCR4D31–3 and all the selected plasmids was over 99% (Supplementary Table 3). Among the selected plasmids, pEC13-1 was from a water sample in a Malaysian pond (Yu et al., 2017), p2018-10-2CC was from a fecal sample of healthy residents in a rural community in Vietnam (Yamaguchi et al., 2020), p1106-IncI2-type and pAH62-1 were from poultry meat samples of Anhui Province, China (Liu et al., 2019; Yin et al., 2021), pHLJ109-11 was from the cecal content of chickens that had died of illness in Heilongjiang Province, China (Li et al., 2020), and the other four plasmids, pColR644SK1, pL889-MCR1, p5CRE51-MCR-1, and p778, were from patients from Switzerland, Zhejiang, China, Taiwan, China, and Ecuador, respectively (Zurfluh et al., 2017; Lin et al., 2019; Loayza-Villa et al., 2020; Liang et al., 2021a). Unfortunately, the patient from which p5CRE51-MCR-1 was isolated died of illness despite treatment. Seven plasmids, including pMCR4D31–3, all contained the *nikA-nikB-mcr-*1-*pap2* structure. Among the other three plasmids, pColR644SK1 and pEC13-1 (from Switzerland and Malaysia, respectively) lacked the *nikA* gene in the *mcr-*1 cassette; p2018-10-2CC only contained the *pap2* gene in sequences flanking the *mcr-*1 gene. Overall, the *mcr-*1 gene in pMCR4D31–3 showed a genetic neighborhood similar to that of the IncI2-type plasmids from various origins, emphasizing the feasibility of horizontal transmission.

## Discussion

In the 21st century, antimicrobial resistance is considered to be one of the most serious global threats to human health. The AMR of *E. coli* is closely associated with the abuse of antibiotics, with a high rate of resistance to antibiotics that have been used for a long time in poultry farming but a very low rate of resistance to antibiotics that are prohibited or restricted in poultry farming (Rhouma et al., 2016; Shen et al., 2016). Poultry farming varies profoundly in different regions of China in terms of scale and method, and the use of antibiotics also varies greatly among regions due to differences in the geographic environment and farming methods. In this study, we found that the antibiotic resistance rate of *E. coli* in poultry farms of Qinghai Province was high, with a resistance rate of 3.47% to colistin and a resistance rate of 1.73% to TIG, and all the *E. coli* isolates in this study were sensitive to MEM. Although poultry farming in Qinghai Province is smaller in scale, shorter in history, and has a more independent poultry farming environment, the drug-resistant *E. coli* in Qinghai is as serious as that in inland areas, suggesting antibiotic abuses in poultry farming in this region. Given that Qinghai Province has a small population and a small number of poultry farms in the western region, and the samples of this study were mostly collected from the eastern areas of the province, the data were limited, and more follow-up monitoring is needed on the drug-resistant *E. coli* strains from chickens in China’s plateau region.

Colistin resistance derived from the horizontal transfer of the *mcr-*1 gene has led to an epidemic risk of colistin-resistant bacteria (Wang et al., 2017). In 8022 stool samples, Zhong et al. found a *mcr-*1 positivity rate of up to 6.2%. It is the diverse genetic mechanisms of the *mcr-*1-carrying IncI and IncHI2/HI2A plasmids that cause the high horizontal transfer rate of *mcr-*1, e.g., the *mcr-*1-related insertion element IS*Apl1* (1,070 bp; Zhong et al., 2018), which was originally discovered in *Actinobacillus pleuropneumoniae* (Tegetmeyer et al., 2008), is the most important mobile element of the IS*30* family mediating the spread of *mcr-*1. The Tn*6330* transposon (IS*Apl1-pap2-mcr*-1-IS*Apl1*) can mediate the horizontal transfer of *mcr-*1, in which IS*Apl1* forms a ring-shaped intermediate in a “cut-and-paste” manner to mediate the spread of *mcr-*1 among different types of plasmids (Girardello et al., 2021). In the QH20-5D4-1 isolate, the transposon element IS*Apl1* was present downstream of *mcr-*1, and the *pap2* gene present upstream of *mcr-*1 encodes the phospholipase closely related to the horizontal transmission of *mcr-*1 (Ma et al., 2021), both playing a very important role in *mcr-*1 mediated bacterial colistin resistance (Choi et al., 2020). Although we did not observe the Tn*6330* transposon unit that mediates the rapid spread of the *mcr-*1 gene in the poultry farms of Qinghai Province, the insertion element IS*Apl1* and the *pap2* gene neighboring *mcr-*1 similarly mobilized the plasmid-mediated horizontal spread of the *mcr-*1 gene.

With the exception of QH20-4D2-1 contig_81 (JAJDKI010000081), all the sequences of the *mcr*-1-carrying *E. coli* isolates contained the *pap2* gene nearby; this gene is associated with colistin resistance and the horizontal transmission of *mcr-*1. However, only in QH20-5D4-1 contig_94 (JAJDKJ010000094), the *pap2-mcr*-1-IS*Apl1* structure was present near *mcr-*1, and Wang et al. noted that the missing IS*Apl1* insertion sequence is a remanent of the frequent shuffling of the *pap2-mcr*-1-IS*Apl1* structure (Wang et al., 2017). It is worth noting that in the QH20-4D2-1 contig_81 sequence downstream to *mcr-*1, the ESBL gene *bla*_CTX-M-64_, which is capable of degrading the third and fourth generations of cephalosporins, was observed. This gene also mediates resistance to amoxicillin, AMP, aztreonam, cefepime, CTX, ceftazidime, ceftriaxone, piperacillin, ticarcillin, and other antibiotics. This gene is located in IS*Ecp1*-*bla*_CTX-M-64_-*Δorf477,* a typical transposon unit, in which IS*Ecp1* and IS*Apl1* are two important insertion sequences for the horizontal transfer of *mcr-*1 (Wang et al., 2017). In 2016, the co-occurrence of *bla*_CTX-M_ and *mcr-*1 in the same *E. coli* strain was first reported (Zhang et al., 2016), and the IncI2-type *E. coli* plasmids simultaneously carrying the two genes have since been continuously reported (Gao et al., 2016; Sun et al., 2016), possibly because the IncI2-type plasmids are also important carriers and transmission vectors of the *bla*_CTX-M_ resistance gene (Wu et al., 2018). The co-presence of *mcr-*1 and *bla*_CTX-M-64_ on the same plasmid may accelerate the spread of the two genes through co-selection (Sun et al., 2016), which provides a way for the bacterium to acquire multidrug resistance more quickly. Consequently, the risk and threat of the epidemic spread of multidrug-resistant bacteria is increased, further suggesting that *E. coli* with the *mcr-*1 gene may have pan-drug resistance (PDR) and cause infections that are difficult to treat clinically. To our knowledge, this study represents the first report on the co-existence of the animal-derived ESBL resistance gene and *mcr-*1 resistance gene in the same *E. coli* plasmid in Qinghai Province.

pMCR4D31–3 shows high homology to the IncI2-type *mcr*-1-carrying plasmids that are geographically distant and different in species. Loayza-Villa et al. discovered the first IncI2-type *mcr*-1*-*carrying *E. coli* plasmid (p778) in Ecuador, which was isolated from a 14-year-old boy. Meanwhile, three non-identical *E. coli* clones isolated from the chickens and dogs of the boy’s family contained similar IncI2-type plasmids, which supports the notion that *mcr-*1 can be horizontally transmitted between animals and humans living in the same environment (Loayza-Villa et al., 2020). When pHNSHP45, a plasmid carrying the *mcr-*1 gene, was first reported, Liu et al. proposed that given that the proportion of *mcr-*1-positive samples in animals is far greater than that of *mcr-*1-positive samples in humans, the *mcr-*1-mediated colistin resistance most likely originated in animals and was then transmitted to humans (Liu et al., 2016). Zurfluh et al. analyzed the *mcr*-1-carrying IncI2-type plasmids isolated from the patient’s urethra, diarrhea patients, and those with travel history to Asian countries (pColR644SK1) as well as from retail poultry meat and turkey meat, and found that the horizontal transfer of the *mcr*-1 gene *via* plasmids is the primary method of spread along the food chain, indicating that the food chain may be an important transmission route of *mcr-*1-carrying plasmids (Zurfluh et al., 2017). It is worth noting that p778 and pColR644SK1 have high homology to pMCR4D31–3 isolated in this study, with similar *mcr-*1 gene cassettes; thus, it is necessary to prevent and control the pMCR4D31-3-mediated horizontal transmission of the *mcr-*1 gene between animals and humans in the same environment and the risk of its spread along the food chain.

## Conclusion

In this study, we provide data on the AMR of chicken-derived *E. coli* in Qinghai Province and the genetic environment of the *mcr-*1 gene and for the first time, generated a complete plasmid profile of the IncI2-type *mcr*-1*-*carrying plasmid from Qinghai, which helps us understand the prevalence of *mcr-*1 in animal-derived *E. coli* in this area. In summary, our study indicates that it is necessary to continuously monitor the AMR of *E. coli* and the prevalence of *mcr*-1 in the plateau region, which is of great significance to understanding and controlling the spread of bacterial antibiotic resistance.

## Data availability statement

The datasets presented in this study can be found in online repositories. The names of the repository/repositories and accession number(s) can be found in the article/Supplementary Material.

## Ethics statement

The animal study was reviewed and approved by Zhejiang Academy of Agricultural Sciences. Written informed consent was obtained from the owners for the participation of their animals in this study.

## Author contributions

BT: conceptualization. HY: funding acquisition. BT, JC, XZ, JM, JuW, XJ, and BD: investigation. JiW, XZ, and BD: methodology. BT and BD: supervision. JM, JiW, and BT: visualization. BT and JiW: writing—original draft. All authors contributed to the article and approved the submitted version.

## Funding

This work was supported by the Key Research and Development Program of Zhejiang Province (2020C02031), the State Key Laboratory for Managing Biotic and Chemical Threats to the Quality and Safety of Agro-products (2010DS700124-ZZ2008, ZZ2102), and the Collaborative Extension Plan of Major Agricultural Technologies in Zhejiang Province (2021XTTGXM03).

## Conflict of interest

The authors declare that the research was conducted in the absence of any commercial or financial relationships that could be construed as a potential conflict of interest.

## Publisher’s note

All claims expressed in this article are solely those of the authors and do not necessarily represent those of their affiliated organizations, or those of the publisher, the editors and the reviewers. Any product that may be evaluated in this article, or claim that may be made by its manufacturer, is not guaranteed or endorsed by the publisher.

## Supplementary material

The Supplementary Material for this article can be found online at: https://www.frontiersin.org/articles/10.3389/fmicb.2022.885132/full#supplementary-material

## Footnotes

^1^ https://cge.cbs.dtu.dk/services/ResFinder/

^2^ https://cge.cbs.dtu.dk/services/VirulenceFinder/

^3^ http://www.mgc.ac.cn/cgi-bin/VFs/v5/main.cgi

^4^ https://cge.cbs.dtu.dk/services/PlasmidFinder/

^5^ https://cge.cbs.dtu.dk/services/MLST/
