# Supplementary material for: Antimicrobial resistance surveillance of Escherichia coli from chickens in the Qinghai Plateau of China
Source: Front Microbiol. 2022 Jul 22;13:885132. doi: 10.3389/fmicb.2022.885132 (PMC9354467; doi:10.3389/fmicb.2022.885132)
Supplement: Supplementary file 1 [file Data_Sheet_1.docx]

**
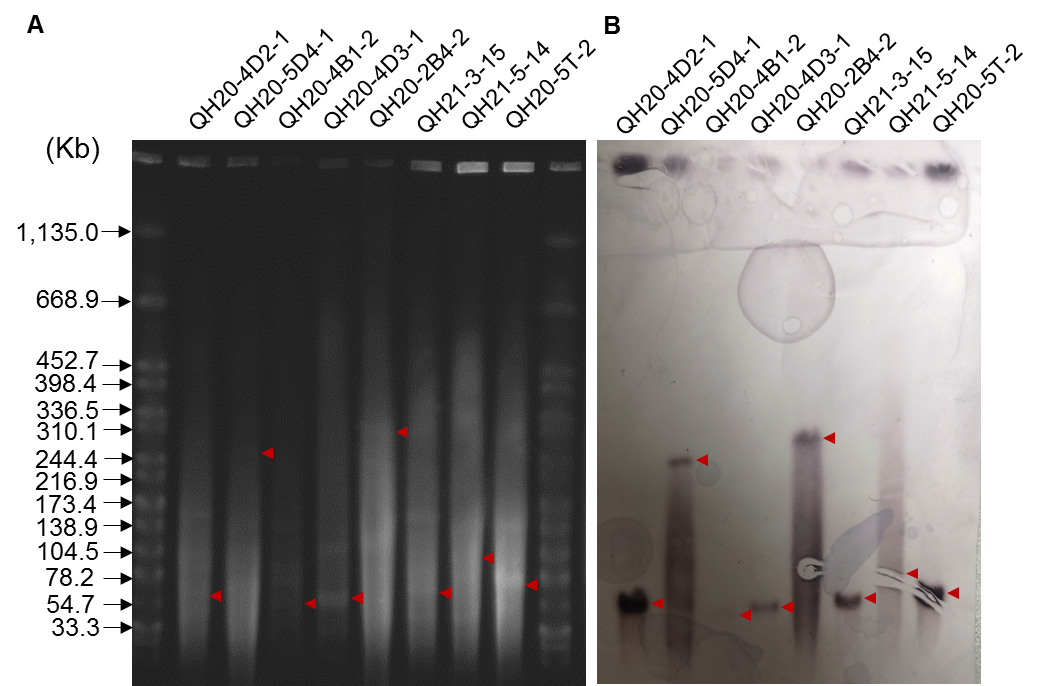
**

**Figure S1.** S1-PFGE (A) and Southern hybridization (B) of *mcr-1*-harboring strains. The plasmids harboring *mcr-1* gene are indicated with red arrows on the fingerprints.

**
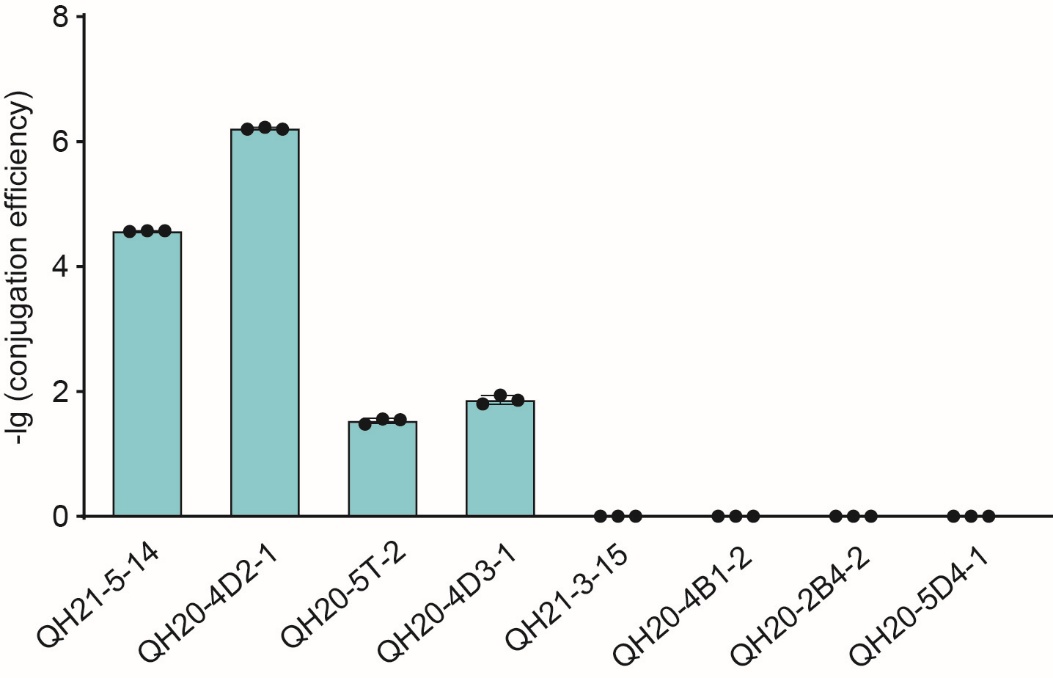
**

**Figure S2.** Conjugation transfer efficiencies of the plasmids harboring *mcr-1* in 8 *E. coli* strains. Transfer efficiency is calculated based on colony counts of the transconjugant and recipient cells in triplicate, and all data points are displayed, along with the mean and standard deviation (error bars).

| year | city | farm | Breeding scale | Sample number | strains |
| --- | --- | --- | --- | --- | --- |
| 2021 | Ledu | F1 | large scale poultry farm | 15 | 14 |
| 2021 | Huangzhong | F2 | large scale poultry farm | 15 | 15 |
| 2021 | Huangzhong | F3 | large scale poultry farm | 20 | 17 |
| 2021 | Huangzhong | F4 | large scale poultry farm | 10 | 10 |
| 2021 | Guide | F5 | family farm | 15 | 14 |
| 2021 | Huangyuan | F6 | family farm | 15 | 15 |
| 2021 | Huangyuan | F7 | large scale poultry farm | 10 | 10 |
| 2021 | Datong | F8 | large scale poultry farm | 10 | 9 |
| 2020 | Maqin | F9 | family farm | 5 | 4 |
| 2020 | Huangzhong | F10* | large scale poultry farm | 210 | 194 |
| 2020 | Huzhu | F11 | large scale poultry farm | 20 | 15 |
| 2020 | Haiyan | F12 | family farm | 15 | 14 |
| 2020 | Tongren | F13 | family farm | 15 | 15 |

**Table S1.** Sampling information of chicken farms in Qinghai plateau.

*The farm consists of four separate areas.

**Table S2.** Isolation and genomic information of 12 strains of colistin-resistant *E. coli*

| **ID** | **Source** | **Host** | **Genome length (bp)** | **Age (d)** | **MLST** | **Accession number** | ***mcr*-1 gene** |
| --- | --- | --- | --- | --- | --- | --- | --- |
| QH20-4B1-2 | Cloacal feces | Broiler | ≈ 5,328,794 | 21 | ST2973 | JAJDKH010000000 | *mcr*-1 |
| QH20-2B4-2 | Cloacal feces | Broiler | ≈ 5,607,133 | 7 | ST43 | JAJDKG010000000 | *mcr*-1 |
| QH20-4D2-1 | Cloacal feces | Broiler | ≈ 5,298,935 | 21 | ST2973* | JAJDKI010000000 | *mcr*-1 |
| QH20-4D3-1 | Cloacal feces | Broiler | 5,436,888 | 21 | ST10 | CP085517-CP085523 | *mcr*-1 |
| QH20-5D4-1 | Cloacal feces | Broiler | ≈ 5,518,478 | 28 | ST93 | JAJDKJ010000000 | *mcr*-1 |
| QH20-2T-1 | Cloacal feces | Broiler | ≈ 5,143,281 | 14 | ST162 | JAJTUM000000000 | none |
| QH20-5T-2 | Cloacal feces | Broiler | ≈ 525,0187 | 28 | ST10 | JAJDKK010000000 | *mcr*-1 |
| QH21-3-15 | Cloacal feces | Layer | ≈ 5,537,387 | / | ST2329 | JAJDKL010000000 | *mcr*-1 |
| QH21-5-14 | Cloacal feces | Broiler | ≈ 5,689,389 | / | ST189 | JAJDKM010000000 | *mcr*-1 |
| QH21-7-8 | Cloacal feces | Layer | ≈ 6,182,052 | / | ST4689 | JAJTUK000000000 | none |
| QH21-2-13 | Cloacal feces | Layer | ≈ 5,353,546 | / | ST295 | JAJTUL000000000 | none |
| QH21-3-13 | Cloacal feces | Broiler | ≈ 5,466,014 | / | ST295 | JAJTUJ000000000 | none |

Notes: * alleles with less than 100% identity were found. / indicates unknown.

**Table S3.** Features of the 9 *mcr*-1-harboring IncI2 plasmids in *E. coli*

| **ID** | **Host strain ID** | ***mcr*-1 harboring plasmid** | **Plasmid size (bp)** | **Acc. No.** | **Country** | **Origin** | **Source** | **Time** | **MLST** | **Inc group** | ***mcr*-1 gene cassette** | **MIC colistin (μg/mL)** |
| --- | --- | --- | --- | --- | --- | --- | --- | --- | --- | --- | --- | --- |
| 1 | 1106 | p1106-IncI2 | 60,960 | MG825374 | Anhui, China | chicken | meat | 2017 | / | IncI2 | *nikA* -*nikB* - *mcr*-1 -*pap2* | / |
| 2 | ColR644SK1 | pColR644SK1 | 60,952 | MF175188 | Zurich, Switzerland | patient | diarrhea | 2016 | ST117 | IncI2 | *nikB* - *mcr*-1 -*pap2* | 4 |
| 3 | L889 | pL889-MCR1 | 63,050 | MZ062604 | Zhejiang, China | patient | fecal | 2016 | ST1011 | IncI2 | *nikA* -*nikB* - *mcr*-1 -*pap2* | 16 |
| 4 | HLJ109 | pHLJ109-11 | 60,960 | MN232196 | Heilongjiang, China | dead broiler chickens | cecal contents | 2016 | ST1011 | IncI2 | *nikA* -*nikB* - *mcr*-1 -*pap2* | 4~16 |
| 5 | AH62 | pAH62-1 | 60,960 | CP055260 | Anhui, China | chicken | heart or liver | 2018 | ST1788 | IncI2 | *nikA* -*nikB* - *mcr*-1 -*pap2* | 8 |
| 6 | 2018-10-2CC | p2018-10-2CC | 60,967 | LC511662 | Vietnam | community healthy human | fecal | 2018 | ST155 | IncI2 | *mcr*-1 -*pap2* | / |
| 7 | 5CRE51 | p5CRE51-MCR-1 | 60,961 | CP021176 | Taiwan, China | patient | urine | 2015 | ST617 | IncI2 | *nikA* -*nikB* - *mcr*-1 -*pap2* | 4 |
| 8 | GN2984 | p778 | 62,311 | MN746292 | Ecuador | patient | peritoneal fluid | 2016 | ST609 | IncI2 | *nikA* -*nikB* - *mcr*-1 -*pap2* | 8 |
| 9 | EC13 | pEC13-1 | 60,218 | CP016186 | Malaysia | pond | water | 2017 | ST410 | IncI2 | *nikB* - *mcr*-1 -*pap2* | 8 |

Notes: / indicates unknown.
